# 

**Published:** 1831

**Authors:** 


### THE MEDICO-CHIRURGICAL REVIEW, AND JOURNAL OF PRACTICAL MEDICINE.

*(NEW SERIES.)*

VOLUME FIFTEEN,

[1st of APRIL to 30th of SEPTEMBER,]

1831.

VOL. XIX. of ANALYTICAL SERIES.

Edited by JAMES JOHNSON, M.D.

PHYSICIAN EXTRAORDINARY TO THE KING,

&c. &c. &c.

Surgeon Gen'l's Office LIBRARY 57454 Washington, D.C.

LONDON:

PUBLISHED BY S. HIGHLEY, 32, FLEET STREET,

AND WEBB STREET, ST. THOMAS'S HOSPITAL,

And all other Booksellers.

W1
ME 75T

PRINTED BY G. HAYDEN,

Little College Street, Westminster.

### BIBLIOGRAPHICAL RECORD;

### 1st of April to 1st July, 1831.

1. An Appendix to the Popular Summary of Vaccination, comprising Experiments with Small-pox Virus upon the Cow, and further Remarks on the Practice of Cow-pox. By John Marshall, Esq. Octavo, sewed, pp. 38. Underwood, 1831.

2. Lecture introductory to a Course of Clinical Surgery, delivered to the Students of the Glasgow Royal Infirmary. By M. S. Buchanan, M.D. one of the Surgeons to the Royal Infirmary. Pp. 25; not published.

☞ *This lecture strongly insists on the importance of clinical instruction, and especially clinical Surgery. This conviction is now becoming universal.*

3. A Supplement to the London, Edinburgh, and Dublin Pharmacopœias; containing a concise View of the Doctrine of Definite Proportions, and its Application to Pharmacy—an Account of the new French Medicines, &c. By D. Spillan, A.M. M.D. one of the Physicians to the Dublin General Dispensary. Octavo, pp. 218, 1830.

☞ *This little work is executed in a masterly manner.*

4. A Translation of the Pharmacopœia of the Royal College of Physicians of London, 1824, with Notes and Illustrations. Second Edition. By Richard Phillips, F.R.S. &c. Lecturer on Chemistry &c. 8vo. 1831.

5. A practical Dissertation on the Waters of Leamington Spa; including the History of the Springs, a new Analysis of their Gaseous Contents, the Rules for drinking the Waters, Bathing, &c. By Charles Loudon, M.D. Physician at Leamington. Third Edition, 1830. Octavo, 5s. 6d.

☞ *We have already, on a former occasion, expressed a very favourable opinion of this volume; and of a third edition, it is unnecessary to go farther than to announce it.*

6. The Surgical Anatomy of some of the principal Vessels of the Head.

☞ *This is on a sheet, with marginal letter-press explanations. It is as large as life, beautifully coloured, and apparently executed (by* Cocks*) with great fidelity.*

7. Lectiones Celsianæ et Gregorianæ; or Lessons in Celsus and Gregory, consisting of Passages from those Authors, syntactically arranged, with copious Observations, &c. for the Use of Medical Students. By William Cross, Teacher of the Classics and Medical Latin. Small 8vo. pp. 169. Wilson, 1831.

☞ *A very useful little vade mecum.*

8. An Essay on the Influence of Temperament in Dyspepsia or Indigestion. By Thomas Mayo, M.D. Octavo, pp. 144, 1831.

9. Introductory Lecture on Midwifery, delivered in February, 1831, at the School of Medicine in Liverpool. By Samuel Malins, M.D. Underwoods, 1831.

10. Treatise on the Excision of Diseased Joints. By James Syme, F.R.S.E. Surgeon of the Edinburgh Surgical Hospital, Lecturer on Surgery, &c. Octavo, pp. 163, with Plates, 7s. 6d. bds. 1831.

11. The Glasgow Medical Examiner, for April, 1831. Edited by S. P. Glen, Esq. Surgeon. No. I.; to be continued Monthly. Octavo, pp. 24.

☞ *One main object of the new periodical is to promote medical reform.*

12. Medical Zoology and Mineralogy; or Illustrations and Descriptions of Animals and Minerals employed in Medicine, &c. No. V. May 1, 1831. By JOHN STEPHENSON, M.D. Price 3s. 6d. each No.

☞ *This work is admirably executed, and deserves the patronage of all classes of the profession.*

13. View of the Pelvis, shewing the natural Size, Form, and Relations of the Bladder, Urethra, Rectum, Uterus, &c. in the Infant and in the Adult, taken from Preparations made for the Museum of the Royal College of Surgeons in Ireland. By JOHN HOUSTON, Curator of the Museum, &c.

☞ *These three plates are done on stone, and are very accurate representations of the parts above-mentioned. Mr. Houston we believe to be one of the best anatomists in these Isles.*

14. An Address delivered before the New York Horticultural Society, on the 8th September, 1829. By J. W. FRANCIS, M.D.

☞ *An eloquent and learned address in favour of horticulture—and, indeed of agriculture.*

15. The Pharmacopœia Universalis; or complete Encyclopædia of the Materia Medica contained in the Pharmacopœias of London, Edinburgh, and Dublin, as well as of all those of Europe and America; and of the Dispensatories, Formularies, and Chemical Works of numerous Writers. Edited by JAMES RENNIE, A.M. Professor of Natural History, King's College, London.

☞ *This Work, which is published in half-crown parts, promises to afford, at a cheap rate, a mine of pharmaceutical, chemical, and botanical knowledge. We have received two parts.*

16. Observations on Distortions of the Spine; with a few Remarks on Deformities of the Legs. By LIONEL J. BEALE, M.R.C.S. Pp. 102. Wilson, May, 1831.

17. Official Papers on the Medical Statistics and Topography of Malaccâ and Prince of Wales' Island, and on the prevailing Diseases of the Tenasserim Coast. By T. M. WARD, M.D. and J. P. GRANT, Esq. Quarto, Penang, 1830.

18. Ornithological Dictionary of British Birds. By CAS. G. MONTAGU, F.L.S. Second Edition, with a Plan of Study, and many new Articles and original Observations. By JAMES RENNIE, A.M. A.L.S. Professor of Natural History, King's College, London, &c. Octavo, pp. 592, 1831.

☞ *This is a treasure of ornithological knowledge, elegant amusement, and useful information. The wood-cuts are numerous and excellent.*

19. The Art of preventing the Loss of Teeth. By JOSEPH SCOTT, Dentist. Octavo, pp. 100, May, 1831.

20. An Introductory Lecture delivered at the College of Physicians and Surgeons of the City of New York, Nov. 1830. By John Beck, M. D., 8vo. sewed.

☞ *An elegant and impressive address.*

21. American Medical Biography; or Memoirs of eminent Physicians who have flourished in America, &c. By JAMES THACKER, M.D. Two Volumes, 8vo. with numerous Portraits. Boston, 1828.

☞ *This work contains a great fund of instructive biography, interesting even in this country, but particularly so beyond the Atlantic.*

22. A Lecture on the Anatomy, Physiology, and Pathology of the Eye. By THOMAS FIRTH, Surgeon. Octavo, sewed, June 1831.

23. The History of Medicine, Surgery, and Anatomy, from the Creation of the World to the Commencement of the 19th Century. By WILLIAM HAMILTON, M.B. In two Vols. 8vo. pp. 419 and 308. 1831.

☞ *Of this work, due notice will be taken in our next Number.*

24. Description of Distinct, Confluent, and Inoculated Small-pox, Varioloid Disease, and Cow-pox; illustrated by Thirteen Plates. By J. FISHER M D. Quarto, pp. 73. Boston, (America) 1829.

☞ *This is a very valuable and meritorious performance.*

N.B. Authors and Publishers are to recollect, that this Journal is re-published in the United States *verbatim*, and, consequently, the BIBLIOGRAPHICAL RECORD is the most extensive advertisement, and the most permanent, which a Work can receive.

